# Molecular Mechanism Underlying ROS-Mediated *AKH* Resistance to Imidacloprid in Whitefly

**DOI:** 10.3390/insects15060436

**Published:** 2024-06-08

**Authors:** Jingjing Li, Chaoqiang Zhu, Yunhao Xu, Haifang He, Chenchen Zhao, Fengming Yan

**Affiliations:** College of Plant Protection, Henan Agricultural University, Zhengzhou 450046, China; jjli@henau.edu.cn (J.L.); chaoqiangzhu6@163.com (C.Z.); 18300699994@163.com (Y.X.); hehaifang55@163.com (H.H.); zhaochen@webmail.hzau.edu.cn (C.Z.)

**Keywords:** *Bemisia tabaci*, imidacloprid, *AKH*, reactive oxygen species, resistance

## Abstract

**Simple Summary:**

As an invasive pest, whitefly causes great losses in agricultural production, and resistance increases in whitefly to chemical insecticides are an important problem in plant protection. Our study revealed that imidacloprid stress inhibits the expression of *AKH*, thereby increasing ROS and the expression levels of the resistance gene *CYP6CM1* and upstream regulatory factors in *Bemisia tabaci*. *AKH* silencing by RNA interference affected the resistance of whiteflies to imidacloprid. These results provide insight into the resistance mechanism in whitefly.

**Abstract:**

Synthetic insecticides used to control *Bemisia tabaci* include organophosphorus, pyrethroids, insect growth regulators, nicotinoids, and neonicotinoids. Among these, neonicotinoids have been used continuously, which has led to the emergence of high-level resistance to this class of chemical insecticides in the whitefly, making whitefly management difficult. The adipokinetic hormone gene (*AKH*) and reactive oxygen species (ROS) play roles in the development of insect resistance. Therefore, the roles of *AKH* and ROS in imidacloprid resistance in *Bemisia tabaci* Mediterranean (MED; formerly biotype Q) were evaluated in this study. The expression level of *AKH* in resistant *B. tabaci* MED was significantly lower than that in sensitive *B. tabaci* (MED) (*p* < 0.05). *AKH* expression showed a decreasing trend. After *AKH* silencing by RNAi, we found that ROS levels as well as the expression levels of the resistance gene *CYP6CM1* and its upstream regulatory factors CREB, ERK, and P38 increased significantly (*p* < 0.05); additionally, whitefly resistance to imidacloprid increased and mortality decreased (*p* < 0.001). These results suggest that *AKH* regulates the expression of resistance genes via ROS in *Bemisia tabaci*.

## 1. Introduction

*Bemisia tabaci* is one of the most important vegetable and ornamental crop pests in the world [1]. It is characterized by a high reproductive capacity and wide host range, with more than 1000 vegetable crops and ornamental plants identified as hosts [2]. Therefore, it is found on all continents except Antarctica [3].

Neonicotinoid insecticides have been used to control whiteflies in the past few decades owing to their low toxicity to mammals [4,5]. This has led to the development of widespread resistance of whiteflies. Imidacloprid, a first-generation neonicotinoid insecticide, is still widely used. Extensive research has focused on the mechanism underlying resistance to imidacloprid [6,7,8], for example, the trans-regulation of *CYP6CM1*, a cytochrome P450 that confers resistance to neonicotinoid insecticides in the whitefly *Bemisia tabaci*, by the mitogen-activated protein kinase (MAPK)-directed activation of the transcription factor cAMP-response element binding protein (CREB) [9]. In addition, recent studies have shown that miR-1517 may be involved in regulating the expression of *CYP6CM1* [10].

One of these hormones, the adipokinetic hormone (Akh), is secreted by the corpora cardiaca (CC) and elicits both carbohydrate and lipid mobilization from the fat body (trehalose from glycogen and diacylglycerol from triacylglycerol, respectively), acting as a functional homolog of glucagon [11,12]. This hormone signals to the G-protein coupled receptor encoded by the Akh receptor gene AkhR to elevate hemolymph lipid and/or trehalose titers and thus redirects energy to the sites and processes where it is required [13,14].

Insecticides can induce a strong oxidative stress response in insects, which is accompanied by the production of a large number of ROS. Excessive ROS will damage insect cells and even lead to insect death [15,16]. The role of Akh in regulating antioxidant systems involved in insect resistance to insecticides has been reported [17]. Velki et al. found that the Akh mature peptide levels in the hemolymph of the firebug *Pyrrhocoris apterus* increased significantly after insecticide treatment [18]. *AKH* regulates the expression of imidacloprid resistance genes by regulating ROS levels [19].

In this study, imidacloprid resistance levels in *Bemisia tabaci* MED were studied. In particular, the molecular mechanism underlying imidacloprid resistance in *B. tabaci* MED was investigated using resistant and sensitive strains, with a focus on the roles of *AKH* and ROS. To evaluate the role of *AKH* in resistance, its response to imidacloprid as well as changes in resistance after *AKH* silencing by RNA interference (RNAi) were evaluated. To evaluate the role of ROS in resistance and changes in ROS in response to imidacloprid, as well as changes in imidacloprid resistance after ROS elimination, were evaluated. Furthermore, the effect of *AKH* on ROS was studied by silencing *AKH*. The results of this study provide insight into the molecular mechanism underlying the imidacloprid resistance of *B. tabaci* MED from the perspectives of ROS and *AKH* and provide a new target for the development of insecticides.

## 2. Materials and Methods

### 2.1. Insect Rearing

A colony of the *Bemisia tabaci* Mediterranean (MED; formerly biotype Q) cryptic species was maintained on cucumber plants, which was not exposed to any insecticide during the culture period and served as a sensitive population in this experiment. Resistant whiteflies were collected from the greenhouse of the Plant Protection Institute of Hunan Academy of Agricultural Sciences (Changsha, Hunan, China, 28°12′ N, 112°59′ E). *B. tabaci* MED was a multi-generation population reared in the presence of imidacloprid (as the resistant population). The culture conditions were 16 h light/8 h dark, 26 ± 0.5 °C, and 75 ± 0.5% humidity.

### 2.2. Bioassay of Insecticide Resistance

The LC50 values and drug resistance of whiteflies after treatment with imidacloprid (pesticide registration no.: PD20131915; product standard no.: GB/T28143-2011; dosage form: emulsifiable oil; imidacloprid 5%, Bayer Crop Science (China), Hangzhou, China) were determined using the agar disk diffusion method. The test device was improved by using a small box with a lid that was ventilated with gauze mesh, leaving a small opening for insects [20]. An appropriate amount of 1% agar was added to the bottom of the lid. The plate was immersed in diluted imidacloprid at various concentrations for 10 s and then placed on the agar after drying naturally. In total, 20 individuals were placed in each small box and the experiment was repeated 10 times. *B. tabaci* MED death was quantified after 48 h, and water was used as the control.

### 2.3. RNA Extraction, cDNA Synthesis, and Real-Time Quantitative Polymerase Chain Reaction

Total RNA was extracted using the TaKaRa RNAiso Plus Kit (Cat# 9109, Lot# AL42064A, TaKaRa, Beijing, China), according to the manufacturer’s instructions. Then, cDNA was synthesized using the TaKaRa Reverse Transcription Kit (Cat# RR047A, Lot# AL21113A, TaKaRa, Beijing, China). To detect the expression of the resistance gene *CYP6CM1*, quantitative real-time PCR (qRT-PCR) was performed using TaKaRa TB GreenR Premix Ex TaqTM II (Cat# RR820A, Lot# AJF2612A, TaKaRa, Beijing, China), according to the manufacturer’s instructions.

### 2.4. RNAi

Cloning and ligation of the adipokinin gene fragment were performed using the pMDTM18-T Vector Cloning Kit (TaKaRa, Cat#6011, Lot#AK91485A, TaKaRa, Beijing, China). After sequencing, double-stranded RNA (dsAKH) was cloned. dsGFP was synthesized using the T7 High Yield RNA Synthesis Kit (YESEN, Code No: 10623ES50, YESEN, Shanghai, China), following the kit instructions. The membrane feeding method (refer to Li et al. 2015) [21] was used to feed dsAKH and dsGFP to sensitive *B. tabaci* MED. The feeding concentration was 500 ng/μL, determined based on a preliminary experiment. After feeding for 48 h, the silencing efficiency and the change in the expression level of the resistance gene *CYP6CM1* were analyzed by qRT-PCR with sequence-specific primers (Table 1). dsGFP was used for comparison.

### 2.5. Determination of ROS Contents in B. tabaci MED

The ROS content in *B. tabaci* MED was measured after treatment and grinding with 250 μL of phosphate buffer (pH 7.4). After full grinding, samples were centrifuged at 13,680× *g* for 5 min, 190 μL of the supernatant was transferred to a 96-well plate, and 10 μL of DCFH-DA (Solarbio, Cat#CA1410, Solarbio, Beijing, China) (10 μmol/L) was added and mixed well. Samples were incubated in a dark room for 1 h [22]. Fluorescence was detected at an excitation wavelength of 488 nm and emission wavelength of 525 nm. The same volume of phosphate buffer and DCFH-DA-adjusted fluorescence value were used as the blank control, and dsGFP treatment was used as the control.

### 2.6. Scavenging Effect of N-acetylcysteine on ROS

The scavenging effect of N-acetylcysteine (NAC) on ROS in insects was evaluated following a previously reported method [19]. In particular, a NAC solution of 1 mol/L was used based on preliminary analyses of concentrations up to 10 mol/L. The volume of liquid feed used was 200 μL, and pure liquid feed was used as the control.

### 2.7. Statistical Analysis

IBM SPSS Statistics 20.0 software (IBM Corp., Armonk, NY, USA) was used for the numerical analyses of all experimental data [23], and independent samples *t*-tests were used for comparisons between groups [24]. The quantitative fluorescence data were analyzed using the 2^−ΔΔCt^ method. The LC50 of imidacloprid was calculated using the SPSS Probit function [25], and then the regression model was fitted and *R*^2^ was calculated. Plots were generated using GraphPad Prism 8.0.2 [26]. The experimental results are expressed as the mean ± standard error.

## 3. Results

### 3.1. Bioassay of Imidacloprid Sensitivity of B. tabaci

We first estimated imidacloprid LC50 values in *B. tabaci* MED. The LC50 of the sensitive *B. tabaci* MED was 51.820 mg/L, and the LC50 of the resistant *B. tabaci* MED was 535.932 mg/L, with a resistance ratio of 10.34. The resistant *B. tabaci* MED was moderately resistant (Table 2).

### 3.2. The Role of AKH in the Response of B. tabaci MED to Imidacloprid

**Figure 1:**
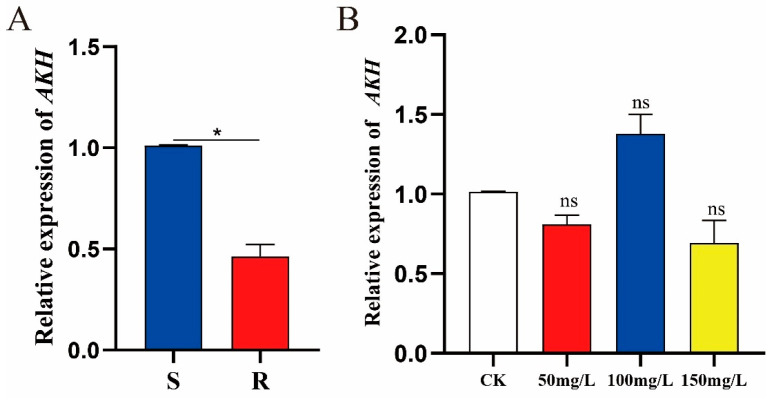
*AKH* expression in sensitive and resistant strains and in response to imidacloprid. Note: (**A**) Comparison of *AKH* expression levels between resistant and sensitive *Bemisia tabaci*, R indicates resistant *B. tabaci*, and S indicates sensitive *B. tabaci*. (**B**) Effect of imidacloprid treatment on the expression level of *AKH* in sensitive *B. tabaci*; the horizontal axis indicates the concentration of imidacloprid, CK represents the control (water). Groups were compared using independent samples *t*-tests, with a significance level of 0.05. Data are presented as the mean ± standard error (* *p* < 0.05, ns indicates insignificant difference).

We compared adipokinin gene expression levels in resistant and sensitive whiteflies. We also evaluated changes in adipokinin in sensitive whiteflies after treatment with imidacloprid. The expression level of *AKH* in resistant *B. tabaci* MED was significantly lower than that in sensitive *B. tabaci* MED (*p* < 0.05) (Figure 1A). After treatment with 50 mg/L imidacloprid, the relative expression level of *AKH* decreased but did not differ significantly from that of the control. Under treatment with 100 mg/L, relative *AKH* expression increased but did not differ significantly from that of the control. After treatment with 150 mg/L imidacloprid, the relative expression of *AKH* decreased but was not significantly different from that in the control (Figure 1B).

### 3.3. Effect of AKH on the Expression of CYP6CM1 and Its Upstream Regulatory Factors

**Figure 2:**
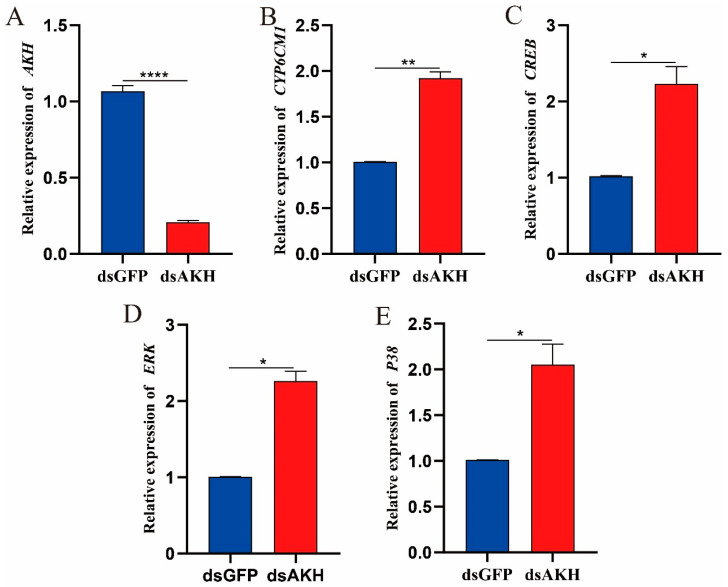
Effect of RNAi silencing targeting *AKH* on the expression of *CYP6CM1* and upstream regulatory factors in sensitive *Bemisia tabaci* MED. Note: (**A**) Expression level of *AKH* after feeding with dsAKH; (**B**) Expression level of *CYP6CM1* after feeding with dsAKH; (**C**) Expression level of *CREB* after feeding with dsAKH; (**D**) Expression level of *ERK* after feeding with dsAKH; (**E**) Expression level of *P38* after feeding with dsAKH. Groups were compared using independent samples *t*-tests, with a significance level of 0.05. Data are presented as the mean ± standard error (* *p* < 0.05, ** *p* < 0.01, **** *p* < 0.0001).

After preliminary experimental data processing and a literature review, we predicted that the lipid-motility hormone is related to imidacloprid resistance in *B. tabaci* MED. Therefore, we used RNAi technology to disrupt *AKH* expression and evaluated the effects on *CYP6CM1* and the three upstream regulatory factors. After feeding double-stranded RNA at a concentration of 500 ng/μL for 48 h by the membrane feeding technique, the relative expression of *AKH* decreased significantly (*p* < 0.0001) (Figure 2A) and the expression of the resistance gene *CYP6CM1* was significantly higher than that in the control (*p* < 0.01) (Figure 2B). The expression levels of *CREB* (*p* < 0.05) (Figure 2C), *ERK* (*p* < 0.05) (Figure 2D), and *P38* (Figure 2E) were significantly higher (*p* < 0.05) in the RNAi group than in the control group, indicating that *AKH* could have an impact on resistance genes and their signaling pathways (Abbreviations).

### 3.4. Effect of AKH Silencing on Imidacloprid Sensitivity

**Figure 3:**
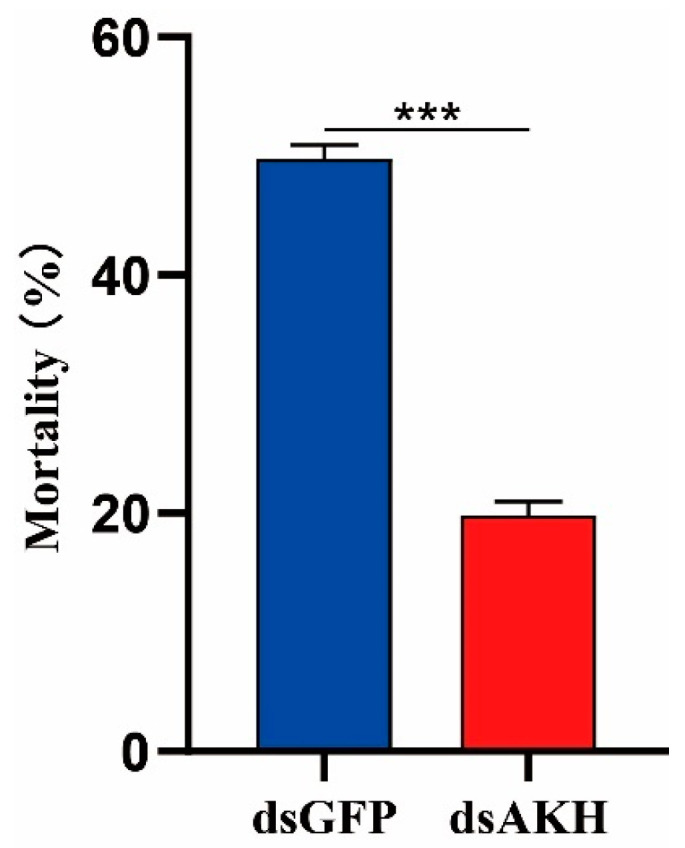
Effect of RNAi silencing targeting *AKH* on the sensitivity of *Bemisia tabaci* MED to imidacloprid. Note: The horizontal axis represents the treatments, and the vertical axis represents the mortality rate of *B. tabaci*. Groups were compared using an independent samples *t*-test, with a significance level of 0.05. Data are presented as the mean ± standard error (*** *p* < 0.001).

Previous analyses indicated that *AKH* can affect the expression of an imidacloprid resistance gene, which is crucial for the metabolism of imidacloprid. Therefore, we evaluated the imidacloprid sensitivity of *B. tabaci* fed dsAKH. After 2 days of feeding with dsAKH, the *B. tabaci* MED was transferred to a bioassay unit and treated with imidacloprid at the LC50 of sensitive *B. tabaci* MED for 48 h. *B. tabaci* MED mortality was significantly lower in the treatment group than in the control group (*p* < 0.001) (Figure 3).

### 3.5. ROS Response to Imidacloprid

**Figure 4:**
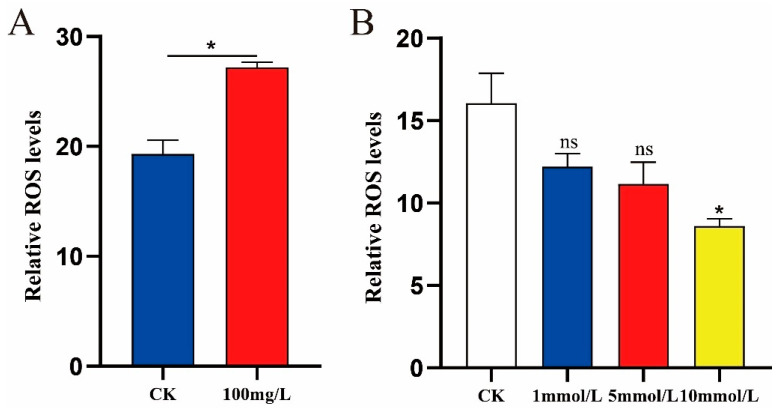
ROS changes in response to imidacloprid. Note: (**A**) ROS response to imidacloprid, CK represents the control (water), and 100 mg/L imidacloprid was applied in the treatment group; (**B**) Changes in ROS contents in resistant *B. tabaci* MED after N-acetylcysteine treatment; CK is the control group (pure liquid feed), and 1, 5, and 10 mmol/L are the concentrations of N-acetylcysteine in the liquid feed. Groups were compared using independent samples *t*-tests, with a significance level of 0.05. Data are presented as the mean ± standard error (* *p* < 0.05, ns indicates insignificant difference).

Insects subjected to insecticide stress will produce a large number of ROS, thus reducing the harmful effects of pesticides [27]. Therefore, sensitive *B. tabaci* MED was treated with imidacloprid to evaluate changes in ROS levels. After treatment with 100 mg/L imidacloprid, the ROS content in *B. tabaci* in the treatment group was significantly higher than that in the control group (*p* < 0.05) (Figure 4A). In addition, to further study the role of ROS in resistance, the ROS scavenger NAC was used to treat resistant *B. tabaci* MED [19]. The results showed that under treatment with 1, 5, and 10 mmol/L NAC, the ROS contents in resistant whiteflies decreased; however, a significant difference was only observed between 10 mmol/L and the control (*p* < 0.05) (Figure 4B). For subsequent studies of resistance mechanisms, a concentration of 10 mmol/L was optimal.

### 3.6. Effects of ROS Scavengers on CYP6CM1 and Its Upstream Regulators

**Figure 5:**
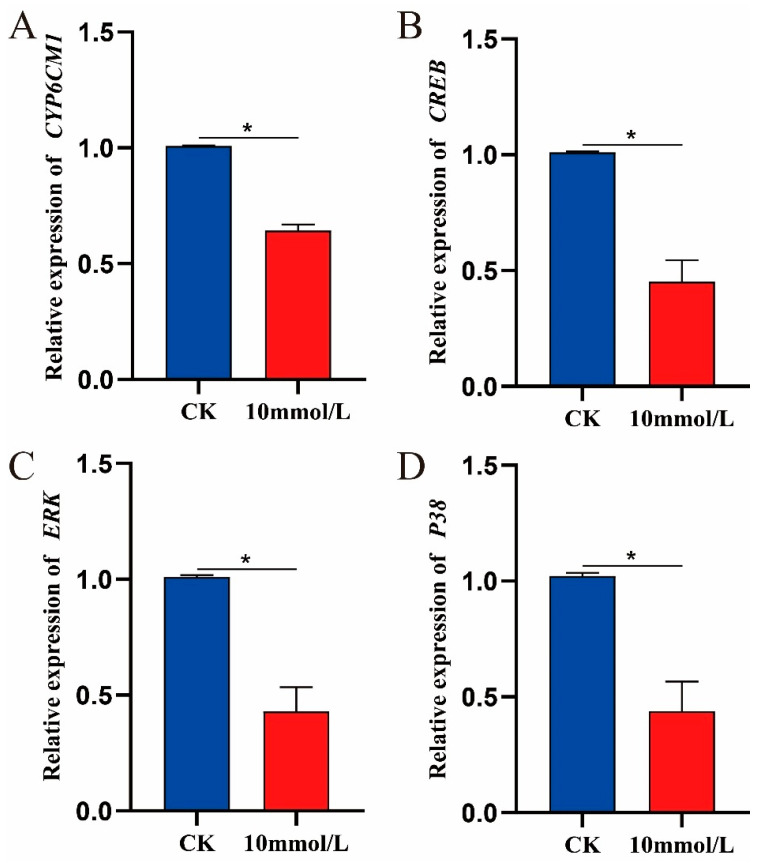
Effect of ROS scavengers on the expression of *CYP6CM1* and upstream regulatory factors in resistant *Bemisia tabaci* MED. Note: (**A**) Expression level of *CYP6CM1* after treatment with 10 mmol/L N-acetylcysteine; (**B**) Expression level of *CREB* after treatment with 10 mmol/L N-acetylcysteine; (**C**) Expression level of *ERK* after treatment with 10 mmol/L N-acetylcysteine; (**D**) Expression level of *P38* after treatment with 10 mmol/L N-acetylcysteine. Groups were compared using independent samples *t*-tests, with a significance level of 0.05. Data are presented as the mean ± standard error (* *p* < 0.05).

ROS levels changed in response to stimulation with imidacloprid, and ROS scavengers significantly reduced ROS levels in resistant *B. tabaci* MED. Therefore, we further evaluated whether ROS could affect the expression of resistance genes. After treatment with 10 mmol/L NAC, the expression level of *CYP6CM1* in resistant *B. tabaci* MED was significantly lower than that in the control group (*p* < 0.05) (Figure 5A). The expression levels of *ERK*, *P38*, and *CREB* were significantly lower than those in the control (all *p* < 0.05) (Figure 5B–D). These results indicate that ROS levels influence the expression of resistance genes and related regulatory factors.

### 3.7. Effect of an ROS Scavenger on Imidacloprid Sensitivity

**Figure 6:**
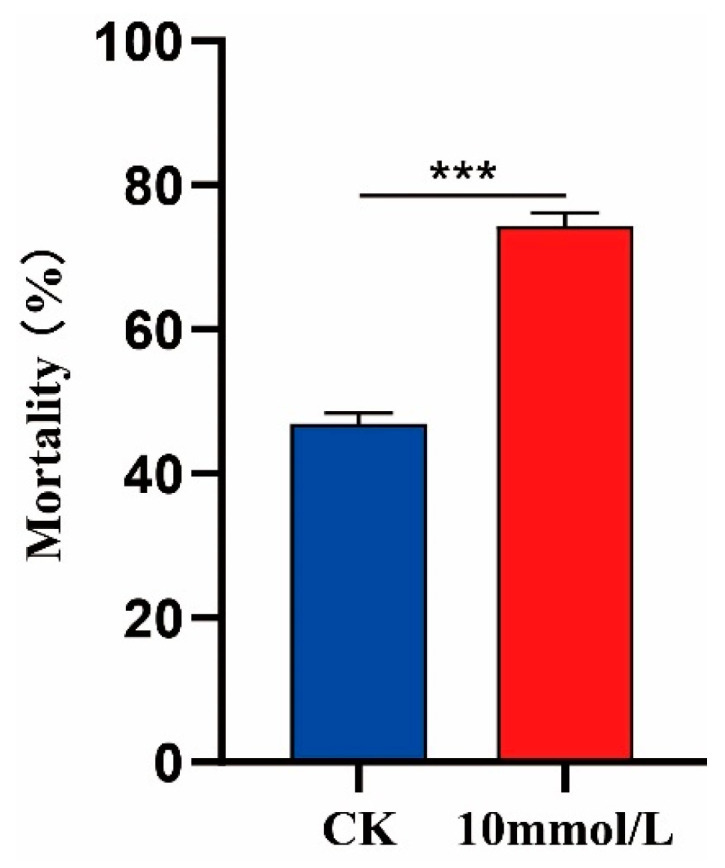
Effect of ROS scavengers on the sensitivity of *Bemisia tabaci* MED to imidacloprid. Note: The horizontal axis represents different treatments, and the vertical axis represents the mortality rate of *B. tabaci* MED. Groups were compared using the independent samples *t*-test, with a significance level of 0.05. Data are presented as the mean ± standard error (*** *p* < 0.001).

The previous analyses showed that ROS can affect the expression of the resistance gene *CYP6CM1*, which is crucial for the metabolism of imidacloprid. Therefore, a bioassay of imidacloprid sensitivity was performed after whiteflies were fed NAC. After 2 days of NAC feeding, whiteflies were treated with imidacloprid at the LC50 dose for 48 h. *B. tabaci* MED mortality was significantly lower in the treatment group than in the control group (*p* < 0.001) (Figure 6).

### 3.8. Effect of AKH on ROS Levels in B. tabaci MED

**Figure 7:**
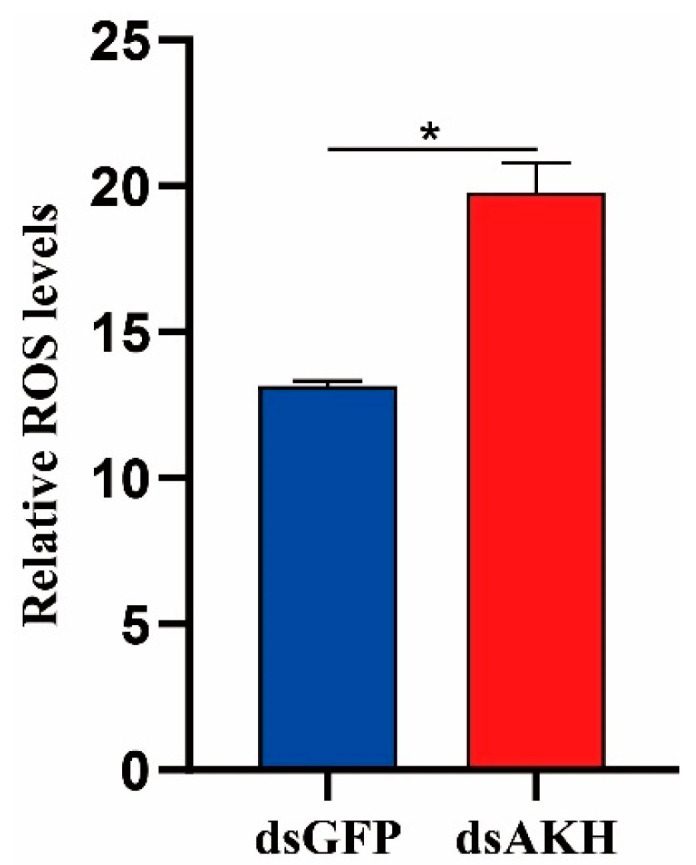
Effect of RNAi silencing *AKH* on the ROS content in *Bemisia tabaci* MED. Note: The horizontal axis represents different treatments, and the vertical axis represents the ROS content in *B. tabaci*. Groups were compared using the independent samples *t*-test, with a significance level of 0.05. Data are presented as the mean ± standard error (* *p* < 0.05).

It has been reported that *AKH* exerts an antioxidant effect in insects [17]. We found that *AKH* and ROS can affect the expression of resistance genes. Therefore, we speculated that *AKH* may regulate the expression of resistance genes by influencing ROS. To evaluate this prediction, levels of ROS in whiteflies were measured after *AKH* was silenced by RNAi. The results showed that the ROS content in the treatment group was significantly higher than that in the control group (*p* < 0.05) (Figure 7), indicating that *AKH* could affect ROS in *B. tabaci* MED.

## 4. Discussion

As a major pest worldwide, *B. tabaci* seriously endangers food security [28,29]. To control *B. tabaci* MED, a large number of chemical pesticides (organophosphorus, pyrethroids, insect growth regulators, nicotinoids, and neonicotinoids.) are used, causing environmental pollution and posing a threat to human health. Pesticide residues are becoming more and more serious; at the same time, whitefly resistance is increasing.

In this study, we found that the *AKH* gene of *B. tabaci* MED contributes to the regulation of resistance to imidacloprid. In particular, *AKH* expression levels were lower in resistant than in sensitive whiteflies After treatment with 50, 100, and 150 mg/L imidacloprid in sensitive *B. tabaci* MED, *AKH* expression exhibited variable levels. This trend was consistent with the results of Tang et al., showing that the *AKH* expression level in the brown planthopper (*Nilaparvata lugens*) is low in resistant strains and decreases after treatment with imidacloprid. However, in this study, the *AKH* expression level increased after treatment with 100 mg/L imidacloprid. It is possible that excess ROS activated the antioxidant system of whiteflies, which has been reported previously [19].

Imidacloprid resistance in *B. tabaci* is mainly caused by the P450 family gene *CYP6CM1* [30]. Consistent with our expectations, we found that *CYP6CM1* expression in *B. tabaci* was significantly increased. Furthermore, *CYP6CM1* is regulated by the MAPK pathway, and the expression levels of the three regulatory factors ERK, P38, and CREB also increased significantly [31]. This suggests that *AKH* may act as a negative regulator of resistance genes. However, the increased levels of resistance genes do not necessarily mean that *B. tabaci* MED tolerance to imidacloprid increased. Therefore, we tested the sensitivity of *B. tabaci* MED to insecticides after silencing *AKH*, revealing that the sensitivity of *B. tabaci* MED to insecticides decreased. This suggests that *AKH* can indeed influence the expression of *CYP6CM1* and thus the resistance of *B. tabaci* MED to imidacloprid.

**Scheme 1:**
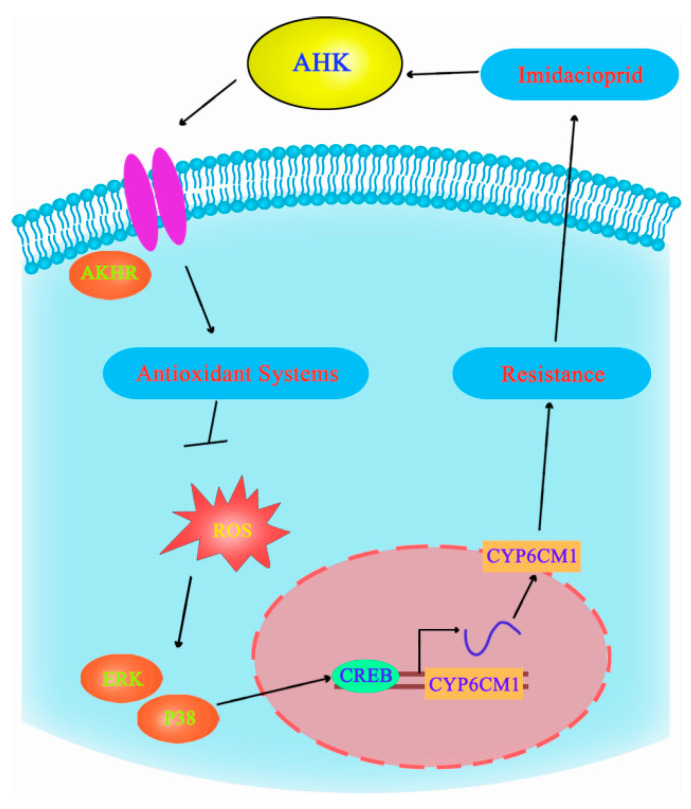
Proposed mechanism of ROS-mediated *AKH* resistance to imidacloprid.

We found that *AKH* exerts a negative regulatory effect on *CYP6CM1* in *B. tabaci* MED. However, the pathways through which *AKH* exerts these effects are still unclear. We speculate that *AKH* exerts its effects through its antioxidant stress function [32,33]. Therefore, we treated the resistant *B. tabaci* MED with the antioxidant NAC and found that the expression levels of the resistance gene *CYP6CM1* and the upstream regulatory factors *ERK*, *P38*, and *CREB* decreased. Moreover, the sensitivity of *B. tabaci* MED to imidacloprid increased (Scheme 1). These results are consistent with those of Tang et al. (2020), who showed that the resistance of the brown planthopper to imidacloprid is mediated by ROS. In another study on the brown planthopper, the expression levels of Akh and AkhR increased significantly under stimulation with chlorpyrifos. Interference with the expression of Akh or AkhR significantly reduced the activity of carboxylesterase and the resistance of the brown planthopper to chlorpyrifos [34]. This suggests that the role of *AKH* in insecticide resistance depends on the type of insecticide and the degree of insecticide stimulation [17,35].

In conclusion, this study demonstrated that exposure to imidacloprid stimulation decreased *AKH* expression, inhibited antioxidant activity mediated by this gene, and increased ROS in *B. tabaci* MED, thereby activating the MAPK signaling pathway and increasing the expression of the resistance gene *CYP6CM1* [31].

## Figures and Tables

**Table 1:** Primers of related genes for qRT-PCR and RT-PCR.

Primer Name	Length (bp)	Sequence (5′→3′)
CYP6CM1-RT-F	24	CACTCTTTTGGATTTACTGCACCC
CYP6CM1-RT-R	22	GTGAAGCTGCCTCTTTAATGGC
CREB-F	22	ACTCAAGGCAGTCTCCAAACCC
CREB-R	22	TTTCTGCTCCGCCTAAATCGTT
P38-F	21	GAACGCCGTCGGAGGATACTT
P38-R	21	TTGGCTCCTTTGAACACTTGC
ERK-F	23	AGATTATTTCCTTCAGCCGATGC
ERK-R	22	GGGCAAGGGCATCTTCAACTAC
Actin-F	20	TCTTCCAGCCATCCTTCTTG
Actin-R	20	CGGTGATTTCCTTCTGCATT
dsAKH-F	47	GATCACTAATACGACTCACTATAGGGCTTGTCGCACAATTCTGGTGT
dsAKH-R	50	GATCACTAATACGACTCACTATAGGGAACTTCTGAACTTCTCACAATCTG
AKH-RT-F	21	CTTGTCGCACAATTCTGGTGT
AKH-RT-R	20	TTGCGCCTCATTCTCGATCA
dsCYP6CM1-F	46	GATCACTAATACGACTCACTATAGGGACTTTTTCAGGGAGGCCATT
dsCYP6CM1-R	46	GATCACTAATACGACTCACTATAGGGGTCGCAGCGTCTCATCAATA

**Table 2:** Virulence equation and resistance multiplicity for *Bemisia tabaci*.

*Bemisia tabaci*	Toxic Regression Equation	Correlation Coefficient	LC50 (mg/L)	Resistance Ratio
Resistibility	Y = −4.093 + 1.5X	0.961	535.932	10.34
Sensibility	Y = −2.699 + 1.574X	0.987	51.820	------

## Data Availability

Data are available on request to the authors.

## Abbreviations

AKH	Adipokinetic Hormone
CREB	cAMP-response element binding protein
ERK	extracellular signal-regulated kinases
P38	p38 mitogen activited protein kinase
ROS	reactive oxygen species
